# Active Starch-Polyester Bilayer Films with Surface-Incorporated Ferulic Acid

**DOI:** 10.3390/membranes12100976

**Published:** 2022-10-06

**Authors:** Eva Hernández-García, Maria Vargas, Amparo Chiralt

**Affiliations:** Research Institute of Food Engineering for Development (IIAD), Universitat Politècnica de València (UPV), 46022 Valencia, Spain

**Keywords:** biodegradable bilayer films, active packaging, PLA-PHBV blend, starch, ferulic acid

## Abstract

Bilayer films of cassava starch-based (with 10% gellan gum) and polylactic (PLA): Poly(3-hydroxybutyrate-co-3-hydroxyvalerate) (PHBV) polyester blend (with 75% PLA) monolayers were obtained by melt-blending and compression-molding, and the subsequent thermocompressing of both monolayers. Ferulic acid (FA) was incorporated into the polyester sheet by spraying and drying. Films were characterized in terms of their microstructure and functional properties throughout two months of storage at 25 °C and 53% relative humidity. The laminates exhibited improved tensile and barrier properties compared to the respective monolayers, which makes them more adequate for food packaging purposes. Surface incorporation of ferulic acid did not significantly modify the barrier and mechanical properties of the films while providing them with antioxidant and antibacterial capacity when applied in aqueous systems, where a complete release of active compounds occurred. The physical properties of the bilayers and layer thermo-sealing were stable throughout storage. Likewise, the antioxidant and antimicrobial active properties were preserved throughout storage. Therefore, these active bilayers represent a sustainable packaging alternative to non-biodegradable, non-recyclable synthetic laminates for food packaging purposes, which could extend the shelf-life of food due to their antioxidant and antibacterial properties.

## 1. Introduction

Active food packaging materials are made with biodegradable polymeric materials from renewable sources, which reduce food waste and are compostable; their use provides a sustainable way to preserve food and increase its shelf-life while using renewable and biodegradable resources to reduce the environmental pollution from plastics and the global dependence on fossil fuel sources. Numerous studies have used biodegradable polymers from a renewable origin as food packaging materials. However, a single biopolymer does not meet the different requirements of food packaging: good mechanical strength and high barrier capacity to water vapor and oxygen or water resistance. In general, polar biopolymers, such as starch, have a good barrier capacity to oxygen and gases but have a low barrier capacity to water vapor and a high sensitivity of their properties to moisture [1]. In contrast, non-polar biopolymers, such as polyesters (e.g., PLA), have good mechanical strength and low gas barrier capacity but good water vapor barrier properties [2,3]. Different strategies have been used to adapt the biopolymer properties to the packaging purposes, such as the use of polymer blends with compatibilizers [2], the incorporation of reinforcing agents, such as cellulose particles or clays [4], or obtaining laminates by combining polymers with complementary properties [5,6,7]. The latter requires good adhesion between the polymer layers, which is compromised by the lack of chemical affinity between polymers and polymer crystallinity [8,9].

The design of active laminates with antioxidant and/or antimicrobial properties involves the incorporation of compounds with these properties into the polymer layer in contact with the food. In the case of high moisture foods where microorganisms proliferate, the contact film must be the most hydrophobic, with low water sensitivity. In addition, the adequate release of the active compound from the film to the food substrate where it has to act is necessary [10]. The compound release and its effectiveness are affected by different factors such as the concentration in the film and its diffusion through the matrix, the minimal inhibitory concentration of the compound for the target microorganism, and the compound interactions with the food substrate that can affect its availability for the active function [10,11].

Ferulic acid is a cinnamic acid derivative present at a relatively high proportion in lignocellulosic plant residues, with high antioxidant [12,13] and antibacterial [14,15] capacity, widely used in the pharmaceutical, food, and cosmetics industry. This compound could be used to obtain active materials for food packaging applications. The incorporation of ferulic acid in PLA matrices, suitable for food contact, provoked positive changes in the properties of the films. However, it presents release problems due to the glassy state of the polymer at the product storage temperature and the subsequent low molecular mobility in the matrix, which prevents the effective release of the active compound into the food systems [16]. The latter results in low activity of the films [17,18], despite the compound’s high antioxidant and antibacterial capacity.

Previous studies [1,19] have demonstrated good properties for food packaging applications of bilayer films constituted by glycerol plasticized cassava starch films with 10% of gellan gum and poly(ethylene glycol), with a molecular weight of 1000 Da (PEG 1000) plasticized PLA films with 25% PHBV. These bilayers, obtained by thermocompression, showed good interlayer adhesion and improved mechanical strength and barrier properties compared to the respective monolayers: lower water vapor permeability than the starch films and lower oxygen permeability than the polyester films. However, incorporating phenolic acids (ferulic, *p*-coumaric, and protocatechuic) into the food contact polyester layer was not effective in providing the films with antibacterial activity due to the limited diffusion of the active compounds in the polyester matrix [18]. Therefore, obtaining active materials with this type of compound and a polyester-based matrix requires a different incorporation strategy that facilitates the compound release and its antibacterial action. Previous studies reported that surface incorporation of ferulic acid onto PLA films, by electrospinning of the PLA-active solutions or by pulverization of concentrated ethanolic solutions of the active compound, gave rise to active films with an adequate compound release for inhibiting the growth of *Listeria innocua* [20].

In the present study, active bilayer films were obtained by thermocompression of cassava starch films (with 10% gellan gum) and PLA films (with 25% PHBV) and by spraying a ferulic acid solution onto the polyester surface, which should be the food contact layer. Films were characterized in terms of their mechanical and barrier properties and bilayer seal strength. The stability of film properties and the layer adhesion throughout storage under controlled conditions of relative humidity and temperature have been analyzed. Likewise, the stability of the superficially incorporated ferulic acid was assessed as a function of the storage time and its potential antioxidant and antibacterial activity in the films.

## 2. Materials and Methods

### 2.1. Materials

Cassava starch (9% amylose) was purchased by Quimidroga S.A. (Barcelona, Spain). Negatively charged low acyl gellan gum KELKOGEL F (MW 3–5 × 10^5^) was supplied from premium ingredients (Murcia, Spain). Amorphous PLA 4060D, density of 1.24 g/cm^3^ and average molecular weight of 106.226 D with 40% of low molecular weight fraction (275 D), as reported by other authors [2], was supplied by Natureworks (Plymouth, MA, USA) and Poly(3-hydroxybutyrate-co-3-hydroxyvalerate) (PHBV) ENMAT Y1000P with 3% hydroxyvalerate was supplied by Helian Polymers B.V. (Belfeld, Holland). The plasticizer, poly(ethylene glycol) with a molecular weight of 1000 Da (PEG1000), was purchased from Sigma-Aldrich (Steinheim, Germany), and the glycerol was obtained from Panreac Química S.L.U. (Barcelona, Spain). Ferulic acid and _D_-limonene were purchased from Sigma-Aldrich (Madrid, Spain). Ethanol 96%, methanol, and magnesium nitrate-6-hydrate (Mg(NO_3_)_2_) were obtained from Panreac Química (Barcelona, Spain). 2,2-Diphenyl-1-picrylhydrazyl (DPPH) was purchased from Sigma-Aldrich (Saint Louis, MO, USA).

Strains of *Listeria innocua* (CECT 910) and *Escherichia coli* (CECT 101) were purchased from the Spanish Type Collection (CECT, University of Valencia, Valencia, Spain). Tryptone Soy Broth (TSB), bacteriological agar, buffer peptone water, Palcam agar base (PAB) enriched with palcam selective supplement for *Listeria,* and Violet-Red Bile agar (VRBA) for *E. coli* were obtained from Scharlab (Barcelona, Spain) and used for antibacterial tests.

### 2.2. Preparation of Bilayer Films

Monolayer films were obtained by melt blending of the film components (50 g of mixture per each batch) using an internal mixer (HAAKE™ PolyLab™ QC, Thermo Fisher Scientific, Germany) and a hot plate press (Model LP20, Labtech Engineering, Bangkok, Thailand), respectively. For preparing the cassava starch monolayer films, starch and gellan gum were mixed (starch:gum ratio of 90:10), using glycerol (0.30 g/g starch) as plasticizer at 130 °C, rotor speed 50 rpm, for 10 min. After processing, the obtained mixtures were cold-milled in a refrigerated batch mill (Model M20, IKA, Staufen, Germany), and the powder was conditioned at 25 °C and 53% relative humidity (RH) for one week. Four g of the conditioned powder were needed to obtain each film (160 mm diameter); this powder was placed on Teflon sheets and preheated at 160 °C for 1 min in the hot plate press and compressed at 160 °C for 2 min at 50 bar, followed by 6 min at 100 bar and a final cooling cycle of 3 min until the temperature reached about 70 °C, as described by other authors [1]. The films obtained were conditioned at 25 °C and 53% RH until they were used to obtain bilayer films.

To obtain the PLA: PHBV monolayer films, the polymers were blended in a ratio of 75:25, using PEG1000 (15 g/100 g polymer) as the plasticizer at 170 °C, rotor speed 50 rpm, for 12 min. After processing, the blends were cold ground in a refrigerated batch mill (Model M20, IKA) and conditioned at 25 °C. Three grams of the conditioned powder were required to obtain each film (160 mm in diameter); this powder was put onto Teflon sheets and preheated at 200 °C for 5 min in the hot-plate press. The films were obtained by compressing at 200 °C for 4 min at 100 bars and a final cooling cycle of 3 min until about 70 °C, as described in previous study [1].

Starch-polyester bilayer films were obtained by thermocompressing the polyester film together with the cassava starch/gellan gum film in the hot plate press (Model LP20, Labtech Engineering) at 180 °C and 100 bar for 2 min and then cooled to 80 °C for 2 min [1]. The bilayer films were stored at 25 °C and 53% RH until analysis.

### 2.3. Superficial Incorporation of Active Solution

FA solution was sprayed on the PLA-PHBV surface of the bilayer films to incorporate the active compound, as described in previous study [20], with some modifications. For this purpose, a 5% solution of FA in 96% (*v*/*v*) ethanol was prepared. The solution was sprayed onto the PLA-PHBV surface of bilayer films of known initial mass (m_i_) using an airbrush (E4182, Elite pro) loaded with the FA solution. Film samples were placed at a vertical distance of 2 cm below the airbrush nozzle and were sprayed at 70 µL/s flow for 6 s. After pulverization, the bilayers were dried at room temperature for 24 h until a constant mass (m_f_) was reached. The mass difference was used to determine the real mass of FA sprayed per surface unit of the film.

The bilayer films with and without superficial FA were conditioned in desiccators with oversaturated Mg(NO_3_)_2_ solutions (53% relative humidity) at 25 °C until the different analyses were carried out immediately after processing (t0) and at 1 (t1) and two months (t2) of storage to evaluate the stability of the material.

### 2.4. Microstructural Analyses

The microstructure of the bilayer films was examined with a Field Emission Scanning Electron Microscope (FESEM Ultra 55, Zeiss, Oxford Instruments, Abingdon, UK). Samples were kept in desiccators with P_2_O_5_ for two weeks at 25 °C to eliminate the film moisture. Then, film samples were cryofractured by immersion in liquid nitrogen to analyze the cross-sections before being placed on support stubs and coated with platinum. The samples were observed at 2 kV.

Optical microscopy at 40× was used to observe the structure of FA crystals formed on the bilayer films after spraying with the FA solution and drying.

### 2.5. Tensile and Barrier Properties

The mechanical behavior of the bilayer films was determined following the standard method ASTM D882 [21] using a universal testing machine (Stable Micro Systems TA-XT plus, Surrey, UK). Eight replicates per formulation were performed. An electronic digital micrometer (Electronic Digital Micrometer, Comecta S.A., Barcelona, Spain) was used to measure the film thickness to the nearest 0.001 mm, at six random positions on the bilayer films, as described in previous study [22]. Film samples of 25 mm × 100 mm were positioned in the tension test clips (model A/TG, Stable Micro Systems, Haslemere, UK) and submitted to a tensile test at 50 mm/min speed until the break. The elastic modulus (EM), tensile strength (TS), and elongation at break (E) were obtained from stress-strain curves.

The oxygen permeability (OP) of the bilayer films was determined according to ASTM D3985-05 [23] methodology using an Oxygen Permeation Analyser (Model 8101e, Systech Illinois, Thame, UK) at 25 °C and 53% RH. The area of the films was 50 cm^2^ and the oxygen transmission rate (OTR) was obtained every 15 min until equilibrium was reached. The measurements were taken in duplicate.

The water vapor permeability (WVP) was determined following the ASTM E96-95 [24] gravimetric method, considering the modification proposed by other authors [25]. For this purpose, three round film samples (3.5 cm diameter) with and without superficial FA were placed in Payne permeability cups (3.5 cm diameter, Elcometer SPRL, Hermelle/s Argenteau, Belgium), filled with distilled water (5 mL) which, in turn, were placed in a chamber with an over-saturated solution of Mg(NO_3_)_2_ to obtain a RH gradient of 53–100, at 25 °C. The cup weight loss was monitored every 1.5 h using an analytical balance (±0.00001 g) until a steady state was reached. WVP was calculated from the slope of the weight loss vs., time curves, as described by other authors [26]. The same procedure was used to analyze the limonene permeability; 5 mL of D-limonene was placed inside the Payne permeability cups and they were stored in a chamber at 25 °C and 53% RH to determine the weight loss vs. time curves, as described in previous study [3]. Three replicates per formulation were performed.

### 2.6. Release Kinetics of Ferulic Acid from the Film in Aqueous Media

The FA content released from the polyester layer in pure water was analyzed to simulate aqueous food, or a culture media, with a_w_ near 1 while evaluating the compound recovery from the films and thus its stability throughout storage time. The amount released as a function of time was determined spectrophotometrically, using a UV-visible spectrophotometer (Thermo Scientific Evolution 201, Waltham, MA, USA). A standard calibration curve was obtained to determine the FA concentration from the absorbance values of differently diluted solutions. FA was quantified by absorbance measurements at 314 nm, using the water extract of a PLA:PHBV film without FA, obtained under the same conditions as blank. The assay was performed in triplicate by immersing 40 mg of film samples in 100 mL of water at 10 °C, without stirring (simulating the contact with a cold storage aqueous food or culture medium), and measurements were obtained every half hour (stirring for 2 min prior to sampling).

Pelegs’ model (Equation (1)) was applied to the experimental points to predict the release kinetics [27]. Since a full release of the incorporated compound was detected (asymptotic value M_∞_/M_o_ near 100, related to k_2_), the k_1_ parameter was optimized with the Excel Solver tool. (Microsoft 365, Microsoft Corporation, Redmond, Washington, USA)

(1) $$\frac{\mathrm{Mt}}{M_{0}}=\frac{t}{k_{1}+k_{2}t}$$

where: M_t_ /M_o_ is the release ratio (in percentage) of active compound, with respect to the amount incorporated into the film, after contact time t; k_1_ and k_2_ are the model constants, where 1/k_1_ is the release rate at the beginning of the process, and 1/k_2_ is the release ratio of active compound at equilibrium (M_∞_/M_o_).

### 2.7. Antioxidant Activity

The antiradical activity of the FA was determined using the free radical 2,2-Diphenyl-1-picryl-hydrazyl (DPPH) method described by other authors [28], with some modifications. This methodology is based on directly obtaining the stable free radical (DPPH) by dissolving the compound in an organic medium. The free radical DPPH exhibits absorbance at 515 nm, which disappears if it accepts a hydrogen radical or an electron from an antioxidant molecule, as described in previous study [29].

Different concentrations of FA were mixed with a 6.22 × 10^−2^ mM DPPH methanolic solution (Abs_515nm_ = 0.7 ± 0.1) to a final volume of 3 mL. The initial concentration of DPPH in the reaction medium was determined from a calibration curve (Abs_515nm_ = 11.32 [DPPH]—0.0038; R^2^ = 0.9992). The antiradical activity was evaluated by the EC_50_ parameter, which is defined as the amount of the antioxidant required to reduce the initial DPPH concentration by 50% once equilibrium was reached. The reaction stability time between FA and DPPH solutions was 135 min after measuring every 15 min. Equation (2) was used to obtain the EC_50_ values.

(2) $$\%\;{\left[DPPH\right]}_{remaining}=\frac{{\left[DPPH\right]}_{t=135}}{{\left[DPPH\right]}_{t=0}}\times 100$$

[DPPH]_t = 135_ is the DPPH concentration value when the reaction is stable, and [DPPH]_t = 0_ is the DPPH initial concentration.

### 2.8. Characterization of the Seal Strength in Bilayers

To determine the seal strength of the bilayer films, these were heat-sealed on one edge, using the same thermocompression conditions used in the preparation of the bilayer films. Dimensions of film strips were 7.62 × 2.54 cm and were sealed in a 2 × 2.54 cm^2^ area. The thermo-sealed films were stored at 53% RH and 25 °C until analyses at different storage times.

Seal strength was determined according to ASTM F88/F88M-15 [30] on ten strips using a universal testing machine (Stable Micro Systems TAXT plus, Surrey, UK). The unsealed edges of the samples were attached to each tension test clips, with a distance between clips of 50 mm, and submitted to an extension test at 200 mm/min. The sealing strength was determined from the average force calculated in 80% of the total force versus distance curve, as described in the standard method, according to Equation (3).

(3) $$\mathrm{Seal}\;\mathrm{strenght}=\frac{\mathrm{Mean}\;\mathrm{Force}\;\left(N\right)}{\mathrm{Film}\;\mathrm{width}\;\left(m\right)}$$

### 2.9. Antibacterial Activity Assessment

The antibacterial capacity of the sprayed bilayer films was analyzed against Gram (-) bacteria using *Escherichia coli* strains (CECT 101, Burjassot, Valencia) and against Gram (+) bacteria, using a *Listeria innocua* strains (CECT 910, Burjassot, Valencia). For this purpose, the strains stored under protective conditions (glycerol 30%) at −25 °C, were regenerated as described by other authors [31], incubating them at 37 °C for 24 h in tryptone soy broth (TSB) (Scharlab, S.L., Barcelona, Spain) until the exponential phase of growth. The revived cultures were diluted appropriately in TSB to obtain a target inoculum of 10^6^ colony forming units (CFU) per mL for *Escherichia coli* and *Listeria innocua.*

Sterile controls were prepared for the assay to confirm the absence of contamination during the process. In addition, inoculated controls were prepared from plates without bilayer films and plates with unsprayed bilayer films. The bilayer films were cut according to the diameter of the plates used (55 mm in diameter), and once placed in a class II laminar flow cabinet (Bio II Advance, Telstar, Terrassa, Spain), they were irradiated with UV light, the same as the rest of the materials used. Subsequently, 10 mL of TSA were poured into Petri dishes (55 mm, diameter) and inoculated with 100 μL of *Escherichia coli* or *Listeria innocua* bacterial suspension (10^6^ CFU/mL) on the plate surface, using an L-form rod to achieve a uniform spread, obtaining a final initial inoculum of 10^4^ CFU/mL. The samples were covered with 55 mm round film samples with and without FA. A non-covered inoculated control was also included.

After that, the samples were incubated for six days at 10 °C. In addition, to determine the initial counts, serial dilutions were made in peptone water and poured onto tryptic soy agar (TSA), and incubated for 24 h at 37 °C.

After six days at 10 °C, the contents of the plates were poured into sterile bags with 90 mL of buffered peptone water and placed for 2 min in a homogenizer (Masticator Paddle blender, IUL Instruments, Barcelona, Spain). After mastication, serial dilutions were performed and poured onto VRB agar (Scharlab, S.L., Barcelona, Spain) for *Escherichia coli* and on plates with Palcam base agar (Scharlab, S.L., Barcelona, Spain) enriched with Palcam selective supplement for *Listeria innocua* (Scharlab, S.L., Barcelona, Spain), as described in previous study [11]. After incubation of the plates at 37 °C for 48 h, the colonies were counted. All determinations were performed in duplicate.

### 2.10. Statistical Analysis

Statgraphics Centurion XVII-64 software (Manugistics Corp., Rochville, MD, USA) was used to perform statistical analyses of the results by analysis of variance (ANOVA). Both a one-way and multifactor ANOVA were used to analyze the influence of composition variables and storage time on the properties of the bilayer films. Homogeneous sample groups were obtained using the LSD method (95% confidence level).

## 3. Results and Discussion

### 3.1. Load and Structure of Ferulic Acid onto the Films

**Figure 1:**
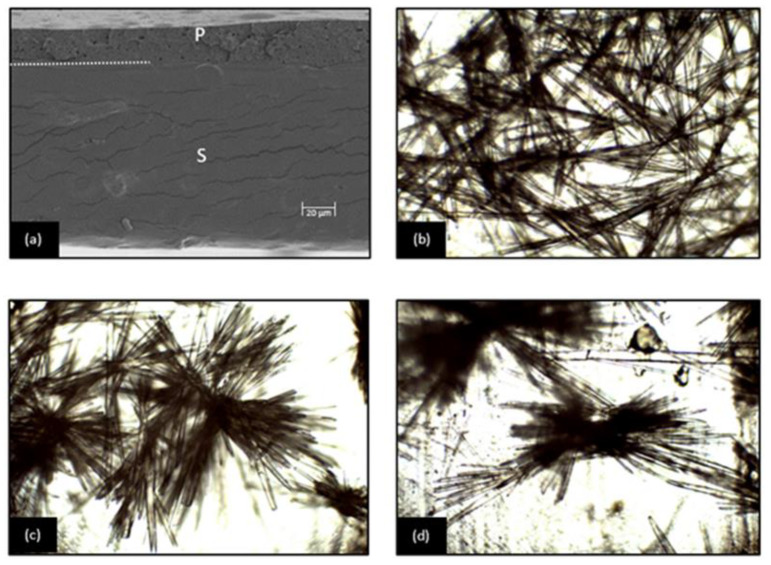
(**a**) FESEM micrograph of bilayer film (cross-section) of cassava starch-based films (S) and PLA:PHBV blend films (P). The polyester (P) and starch (S) sheets are marked, as well as the layer interface (dashed line). (**b**–**d**) Light microscopy images (40×) of FA crystalline formations onto the PLA surface of bilayers after spraying and drying the compound ethanolic solution.

Figure 1a shows the cross-section microstructure of laminate, obtained by FESEM, with the starch-based and PLA:PHBV layers well adhered at the interface, as reported in a previous study [1], where the better adhesion properties of cassava starch-based films with the PLA-PHBV blend was observed. The polyester sheet was much less thick than the starch sheet, mainly due to the lower ratio of PLA in the laminate (polyester-starch mass ratio of 3:4) since a different polymer mass was required to obtain 16 cm in diameter films due to the different flowability of the materials. However, the thickness ratio of polyester:starch layers in the bilayer films was about 1:3.5, which agrees with the higher flowability of the polyester layer than that of starch-based that also contributed to the higher thinning of the polyester layer during the thermo-sealing step of the bilayers. The thickness of the bilayers was about 230 mm (Table 1), in which the polyester sheet had about 50 μm, whereas the starch-based layer had about 180 μm.

The amount of FA incorporated onto the polyester surface by spraying and drying, estimated from the mass difference between the samples before and after the compound application, was about 0.52 mg/cm^2^ of film. The target surface concentration of FA in the films can be easily modulated by controlling the spraying time, flow rate, and compound concentration in the sprayed solution. The method was very effective in loading the films with a large amount of active compound. At the same time, it is easily implementable on an industrial scale without submitting the thermosensitive active compound to high temperatures, regardless of the thermal processing of the polymeric material. The obtained value was enough to reach the minimal inhibitory concentration (MIC) of the tested bacteria in the culture medium if the compound was fully released. In fact, the films used for the antibacterial test (23.76 cm^2^) could deliver to the culture medium (10 mL) an active concentration of 1250 mg/L, which is enough to overcome the MIC of this compound for *L. innocua* and *E. coli* (700 and 800 mg/L, respectively [18]), thus inhibiting the bacterial growth, as previously observed by other authors [20] for PLA films with FA incorporated by the spraying method.

Pulverized solutions of FA on the polyester surface gave rise to the formation of crystalline structures, which were well attached to the film surface after the solvent evaporation, as can be observed in Figure 1b–d. FA crystalline formations were similar to those observed by other authors [20] on the PLA film surface. Chen et al. [32] reported that FA crystallizes in needle-like structures in oversaturated solutions. Therefore, the fast solvent evaporation in pulverized films promoted the oversaturation of the compound on the film surface and its crystallization on the film surface, forming well-adhered crystalline structures. The initial attachment of FA to the PLA surface can be attributed to PLA’s electric polarization and static electricity [33]. Once a small crystal is attached, its growth can occur if exposed to an oversaturated stream.

Due to the crystallization, no homogeneous distribution of FA could be observed on the film surface (Figure 1b–d), but agglomerated crystalline formations of differing sizes were distributed on the film surface. The crystal sizes could affect the release rate of the active compound since the compound’s solubilization rate may be affected by the crystalline structure and crystal sizes [34]. The crystal size distribution could be modulated by modifying the spraying conditions, such as solvent and compound concentration, nozzle type, or flow rate [34,35].

### 3.2. Tensile and Barrier Properties of the Bilayers and their Stability throughout Storage

Table 1 shows the tensile parameters and water vapor, oxygen barrier properties, and permeability to limonene (as a model of aroma compounds) for both FA-coated and uncoated bilayers at different storage times under controlled temperature and relative humidity conditions. The values of elastic modulus, tensile strength, and elongation at break of the newly prepared, uncoated bilayer were similar to those previously reported for this kind of bilayer [1,19]. As expected, no significant changes were provoked by the coating with FA crystals that did not affect the films’ mechanical behavior. Both bilayers exhibited EM and TS values near those of the polyester sheet (780 MPa and 15 MPa, respectively) and the starch monolayers (680 MPa and 18 MPa, respectively), as previously reported by other authors [1]. However, the extensibility of the bilayers was in the range of that of the polyester sheet, which limited the stretchability of the bilayers. No significant changes occurred in the tensile parameters throughout the storage time of both bilayers, which account for the high mechanical stability of the material under storage. Interlayer migration occurs during the layer thermo-adhesion step at high temperatures, which modifies the tensile behavior as compared to that expected from the individual layers [1]. However, this process seems to be inhibited at a lower temperature (25 °C), and no further tensile behavior modification occurred during the storage time. Starch retrogradation [36] and PLA physical aging [37] could also modify the tensile behavior of the films throughout storage. However, no changes that reflect these phenomena were observed under the storage conditions. Cassava starch was less sensitive to retrogradation due to its lower amylose content [36].

Barrier properties (WVP and OP) were also in the previously reported range for these kinds of bilayers [1,19]. A significant decrease in WVP values was observed for the bilayer assembly as compared to the corresponding values of the starch monolayer (11 g·mm·kPa^−1^·h^−1^·m^−2^, [1]), as previously observed in other starch-polyester laminates [26,38] due to the barrier effect of the polyester layer. Likewise, OP significantly decreased as compared to the value of the polyester (410 × 10^−14^ cm^3^·m^−1^·s^−1^·Pa^−1^, [1]) due to the oxygen barrier effect of the starch sheet. Thus, a global improvement of the barrier capacity to water vapor and oxygen of the material was obtained with the bilayer assembly. Likewise, the aroma barrier capacity of the bilayers, evaluated through the limonene permeability, was lower than that of pure PLA films [3,39] but higher than the values reported for cassava starch films [40].

The permeability values were not significantly affected by the bilayer coating with FA nor by the storage time, which, as occurred with the tensile parameters, indicates the great stability of the material throughout at least two months. Therefore, the bilayer coating with FA by spraying with a compound ethanolic solution did not significantly modify the functional properties of the film as packaging material while providing it with antioxidant/antimicrobial potential, as discussed in the next section.

### 3.3. Stability of the Bilayer Thermo-Sealing

Thermo-sealing of polymer sheets is a complex process affected by different factors, such as polymer compatibility, interfacial energy, and polymer crystallinity [5,41]. Once the polymer sheets are thermo-sealed, the lack of chemical affinity and structural changes occurring during storage can lead to their detachment, losing their packaging properties. Therefore, the analysis of the seal strength at different storage times is relevant to ensure the material’s functionality [9].

**Figure 2:**
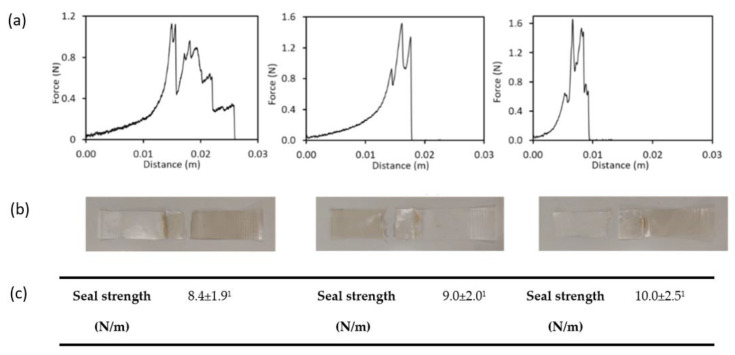
Typical behavior of seal profile of the starch-polyester bilayer films at different storage times (t0, t1, and t2). (**a**) Force-distance curves. (**b**) Image of the sheet separation mode: delamination and (**c**) mean values and standard deviation of seal strength (N/m). Different superscript numbers indicate significant differences between the seal strength values at the three storage times (*p* < 0.05).

Figure 2 shows the force-distance curves of the uncoated bilayers since no differences are expected for the superficially coated bilayers where the crystallized active compound on the polyester external surface would not affect the sealing force. A delamination detachment mode was observed for all tested bilayers, as shown in Figure 2. The obtained values for the seal strength were lower than those reported for PLA-starch seals [9,42,43,44] but remained without significant changes throughout storage time. Differences could be attributed to the distinct sealing conditions, the presence of PHBV dispersed phase in the PLA matrix, and gellan gum dispersed into the starch matrix, which will modify both the PLA and starch adhesion properties. Starch-polyester assemblies exhibited high sealing, while samples exhibited delamination, thus indicating that strong adhesion forces are established between both polymers that lead to the sheet fracture and no to net layer separation. Delamination rather than adhesive peeling indicates strong interfacial bonding of the films [45]. The good adhesion properties of starch-PLA, as compared to other polyesters, were attributed to the lower tendency to self-association of PLA and its lower crystallinity [8]. Likewise, PLA in the obtained laminates is an amorphous polymer with a fraction of low molecular weight oligomers [2], which may contribute to increasing its polarity and chemical affinity with starch molecules at the interface, thus favoring the interlayer adhesion and stability throughout time. In fact, storage time did not significantly affect the sealing force of bilayers, thus demonstrating that thermo-sealing was also stable throughout at least two months.

### 3.4. Stability of Superficially Incorporated Ferulic Acid and Antioxidant and Antimicrobial Properties of the Films

**Figure 3:**
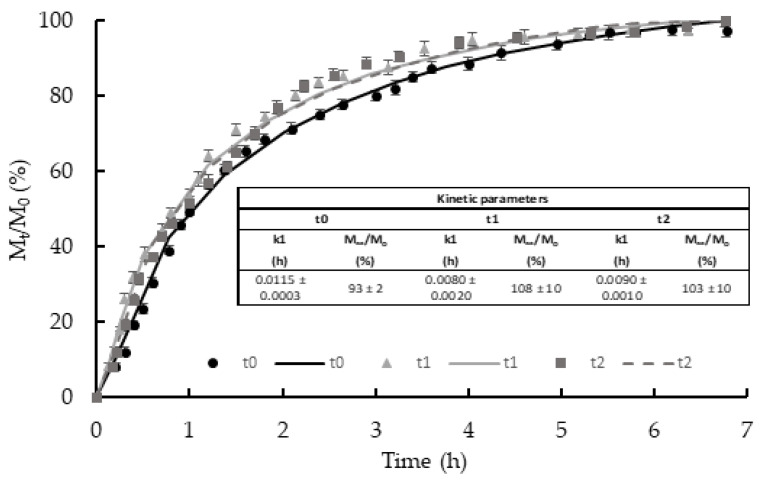
Values of M_t_/M_o_ (ratio of the mass of FA released at each time with respect to the initial mass in the film) as a function of time (experimental points and fitted Pelegs’ model (lines)) for FA coated films stored for 0, 1 and 2 months. The embedded table shows the values of k1 Peleg’s parameter and the experimental values of M_∞_/M_o_ (% released at equilibrium). Mean values and standard deviation.

The stability of FA incorporated on the film surface is also critical to ensure active properties of the bilayer throughout time. The coated bilayers were submitted to a FA release assay when stored for different periods (0, 1, and 2 months) to verify FA stability. The release test was carried out in distilled water to simulate aqueous systems, such as moist foods or culture media, with a_w_ near 1, where microbial growth can easily occur. Figure 3 shows the release kinetics of incorporated FA in the newly prepared and stored bilayers, expressed as the amount released at each time with respect to the initially incorporated on the bilayer. After about a six-hour contact time, a total recovery of the initially incorporated FA was obtained for all films stored for different times (M_∞_/M_o_ near 100 in all cases, without significant differences due to the storage time). The latter indicates that no oxidation or degradation of the compound occurred during the film storage, thus maintaining its potential antioxidant/antimicrobial capacity. Likewise, the initial release rate (related to the 1/k_1_ value) had no significant differences between samples. The release rate could be affected by the crystal sizes of FA that could rise during storage through the recrystallization phenomenon.

The antioxidant power of the compound was estimated through the DPPH radical scavenging capacity determined as the EC_50_ value. This value was 0.172 ± 0.003 mg AF/mg DPPH, which is in the range of the most potent antioxidant compounds, such as ascorbic acid or d-tocopherol (0.12 and 0.26 mg compound/mg DPPH, respectively, as reported by other authors) [28]. Therefore, considering the release capacity of the compound in aqueous media, an adequate antioxidant capacity of the coated films could be expected in aqueous systems, with no losses in this capacity of the film during storage for at least two months.

**Figure 4:**
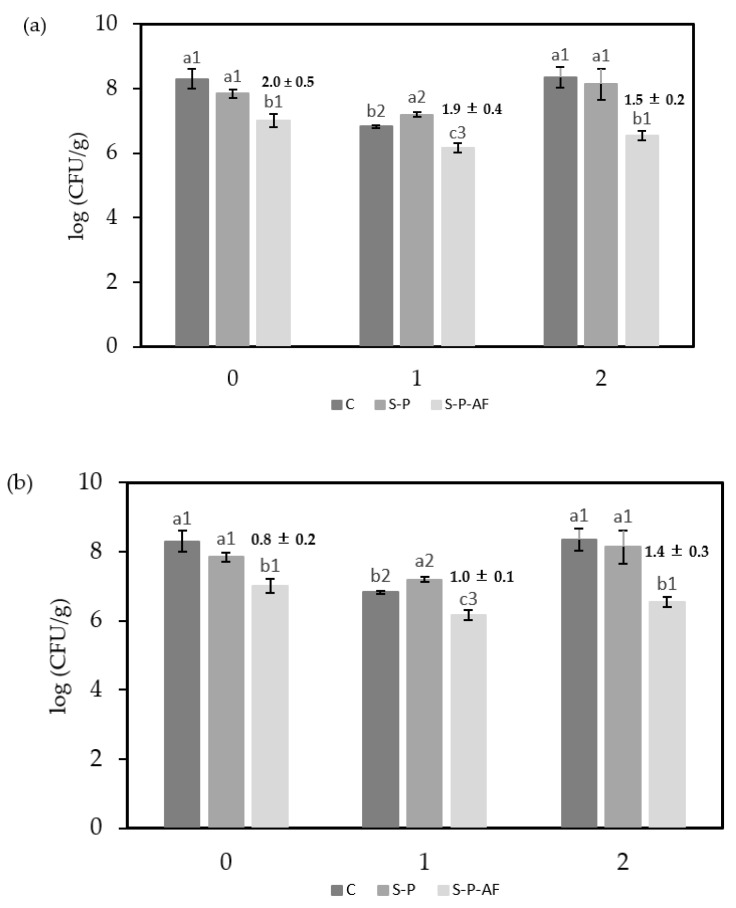
Microbial counts for (**a**) *Listeria innocua* and (**b**) *Escherichia coli* obtained after 6 days of incubation at 10 °C in TSA medium. Uncovered control plates (C), and covered plates with starch-polyester bilayer films: films without FA (S-P) and with FA (S-P-AF). Different superscript letters (a–c) indicate significant differences among formulations (*p* < 0.05). Different superscript numbers (1–3) indicate significant differences between the three storage times (*p* < 0.05). The numeric label on the S-P-AF bars gives the difference between the Log CFU of S-P-AF active film with respect to the S-P film.

Antibacterial properties of newly obtained films and those stored for different times were analyzed with gram-positive (*L. innocua*) and gram-negative (*E. coli*) bacteria. Figure 4 shows the bacterial counts obtained after six days of incubation of an inoculated plate covered or not with the FA-coated and with the non-coated bilayers stored for 0, 1, and 2 months at 10 °C. Significant bacterial growth inhibition was obtained for FA-coated bilayers with respect to those without FA, thus indicating an effective release of FA into the culture media. About 2 log CFU of growth inhibition was obtained for *L. innocua*, without remarkable differences associated with the film storage time, as expected from the preservation of FA in the films during storage. The latter implied a significant antibacterial action from the microbiological point of view, while lower growth inhibition (about 1 log CFU) was obtained for *E. coli*, without significant differences between films stored for different times. Previous studies also observed a higher antibacterial effect of FA against gram-positive than against gram-negative bacteria [46,47]. Gram-negative bacteria, such as *E. coli,* are expected to be more resistant to antimicrobials, as their outer membrane is very challenging for small molecules to cross [48].

## 4. Conclusions

Bilayer films made with cassava starch-based (with 10% gellan gum) and PLA-based (with 25% PHBV) monolayers exhibited improved tensile and barrier properties compared to the respective monolayers, which makes them more than adequate for food packaging purposes. Surface incorporation of FA onto the PLA-based food contact layer by spraying and drying did not significantly modify the functional properties of the films while providing them with antioxidant and antibacterial capacity when applied in aqueous systems where a complete release of active compounds occurred. The physical properties of the bilayers and layer thermo-sealing were stable throughout at least two months of storage at 25 °C and 53% relative humidity. Likewise, the film’s active properties were maintained throughout storage since FA did not degrade in the well-adhered crystallized forms on the polyester blend film surface. Therefore, these kinds of bilayers represent a sustainable packaging alternative to non-biodegradable, non-recyclable synthetic laminates for food packaging purposes, useful to extend the shelf-life of food due to their antioxidant and antibacterial properties.

## Figures and Tables

**Table 1:** Tensile properties (elastic modulus: EM, tensile strength: TS, and deformation at break: %E) and barrier properties (water vapor permeability (WVP), oxygen permeability (OP), and limonene permeability (LP)) of starch-polyester bilayer films (S-P) and FA coated starch-polyester bilayer films (S-P-AF) at time zero and after 1 and 2 storage months. Mean values and standard deviation.

Bilayer Film	S-P				S-P-AF	
	t0	t1	t2	t0	t1	t2
Thickness (µm)	239 ± 10 ^a1^	235 ± 12 ^a1^	230 ± 18 ^a1^	231 ± 15 ^a1^	234 ± 14 ^a1^	228 ± 10 ^a1^
EM (MPa)	871 ± 21 ^a1^	850 ± 47 ^a1^	863 ± 18 ^a1^	850 ± 10 ^a1^	887 ± 53 ^a1^	875 ± 25 ^a1^
TS (MPa)	18.0 ± 2.0 ^a1^	16.0 ± 2.0 ^a1^	17.0 ± 1.0 ^a1^	17.0 ± 3.0 ^a1^	18.0 ± 4.0 ^a1^	19.0 ± 3.0 ^a1^
E (%)	3.0 ± 0.5 ^a1^	3.0 ± 1.0 ^a1^	2.0 ± 0.5 ^b1^	3.0 ± 1.0 ^a1^	3.0 ± 1.0 ^a1^	3.5 ± 0.4 ^a1^
OP × 10^14^ (cm^3^⋅m^−1^⋅s^−1^⋅Pa^−1^)	1.25 ± 0.08 ^a1^	1.30 ± 0.03 ^a1^	1.28 ± 0.01 ^a1^	1.27± 0.02 ^a1^	1.28 ± 0.02 ^a1^	1.30 ± 0.04 ^a1^
WVP (g⋅mm⋅kPa^−^^1^⋅h^−1^⋅m^−2^)	1.6 ± 0.2 ^a1^	1.7 ± 0.1 ^a1^	1.4 ± 0.2 ^a1^	1.5 ± 0.3 ^a1^	1.3 ± 0.2 ^b1^	1.7 ± 0.4 ^a1^
LP (g⋅mm⋅kPa^−^^1^⋅h^−1^⋅m^−2^)	1.2 ± 0.2 ^a1^	1.3 ± 0.1 ^a1^	1.5 ± 0.2 ^a1^	1.1 ± 0.4 ^a1^	1.0 ± 0.1 ^b1^	1.3 ± 0.3 ^a1^

Different superscript letters (a,b) indicate significant differences among formulations (*p* < 0.05). Different superscript numbers indicate significant differences between the three storage times (*p* < 0.05).

## Data Availability

Data is contained within the article and also available on request.
